# The Circ-CYP24A1-miR-224-PRLR Axis Impairs Cell Proliferation and Apoptosis in Recurrent Miscarriage

**DOI:** 10.3389/fphys.2022.778116

**Published:** 2022-03-03

**Authors:** Yan Su, Jiani Xu, Rufei Gao, Xiaoli Liu, Taihang Liu, Cong Li, Yubin Ding, Xuemei Chen, Junlin He, Xueqing Liu, Chunli Li, Hongbo Qi, Yingxiong Wang

**Affiliations:** ^1^Laboratory of Reproductive Biology, School of Public Health and Management, Chongqing Medical University, Chongqing, China; ^2^Joint International Research Laboratory of Reproduction & Development, Chongqing Medical University, Chongqing, China; ^3^Department of Clinical Laboratory, Chongqing Health Center for Women and Children, Chongqing, China; ^4^Department of Obstetrics and Gynecology, The First Affiliated Hospital of Chongqing Medical University, Chongqing, China; ^5^Department of Family Planning, Chongqing Health Center for Women and Children, Chongqing, China

**Keywords:** recurrent miscarriage, circRNA, proliferation, apoptosis, decidualization

## Abstract

**Aim:**

Recurrent miscarriage (RM) is associated with numerous clinical factors. However, some RM occurred without specific factors. It has been revealed that some molecules such as hormones, miRNAs, and transcription factors are involved in RM by regulating proliferation, apoptosis, etc. However, the mechanism of RM has yet to be identified clearly. Circular RNAs (circRNAs) are a class of endogenous non-coding RNAs that often act as sponges for miRNAs or binds to proteins involved in biological processes. However, the functional role of circRNAs in the uterine decidua of patients with early RM is still unclear. In this study, we aimed to investigate the mechanisms of circ-CYP24A1 in RM.

**Methods:**

The Dual-Luciferase Activity Assay was designed to analyze the bonding between circ-CYP24A1 and miR-224, and miR-224 and prolactin receptor (PRLR) mRNA 3′UTR. *In situ* hybridization (ISH) and immunohistochemistry (IHC) were used to observe the expression of circ-CYP24A1 and PRLR in the decidua. Rescue experiments were performed to investigate the regulating effects of circ-CYP24A1, miR-224, and PRLR. Western blotting was conducted to test the expression level of PRLR. The proliferation and apoptosis-related markers in Ishikawa cells were analyzed using CCK8, immunofluorescence staining, and the terminal deoxynucleotidyl transferase-mediated dUTP-biotin nick end labeling (TUNEL) assay.

**Results:**

In this study, based on the microarray analysis data, we identified a high level of circ-CYP24A1 and PRLR in the decidua of patients with early RM. Based on the bioinformatics prediction, the binding relationship between circ-CYP24A1 and miR-224, as well as miR-224 and PRLR, were verified. Functional experiments demonstrated that circ-CYP24A1 regulated proliferation and apoptosis by binding to and inhibiting miR-224, resulting in increased PRLR expression. Taken together, this study provides new insights into the mechanism of RM.

**Conclusion:**

In this study, we found that circ-CYP24A1 plays a role in RM by impairing the balance of cell proliferation and apoptosis by sponging miR-224, thereby regulating PRLR.

## Introduction

Recurrent miscarriage (RM) is defined as three or more consecutive miscarriages within 24 weeks. It is recognized as the most common complication in pregnancy, accounting for 1–5% of all pregnancies and 15–20% of miscarriages (Rai and Regan, 2006; Dean et al., 2018). Miscarriage occurring before the 12th week of pregnancy is defined as early RM. Early RM is associated with numerous clinical factors, such as infectious diseases, abnormal uterine structure, poor parental fitness, endocrine dysfunction, thrombosis tendency, and autoimmune diseases (Stephenson and Kutteh, 2007; Ford and Schust, 2009). However, early RM sometimes occurs without any detectable reason, seriously affecting patients’ physical and mental health.

A successful pregnancy requires a well-prepared endometrium and an activated blastocyst (Jia-Rong et al., 2009). Pregnancy is a complex, irreversible process consisting of embryo implantation (Kitazawa et al., 2020), endometrium decidualization (Salker et al., 2010), and placentation (Carter, 2020). In humans, the endometrium undergoes decidualization to become transiently receptive, preparing for embryo implantation during the mid-luteal phase of the menstrual cycle (Cha et al., 2012). Decidualization is driven by timely proliferation, apoptosis, and differentiation of endometrial stromal cells and is marked by high expression of prolactin (PRL) and insulin growth factor binding protein-1 (IGFBP1) (Fabi et al., 2017; Ochoa-Bernal and Fazleabas, 2020). RM has been associated with abnormal proliferation, apoptosis, and differentiation of endometrial stromal cells (Diniz-da-Costa et al., 2021; Meng et al., 2021). Hong et al. (2018) showed that the microRNA miR-378a-3p plays a role in RM by regulating the proliferation and apoptosis of decidual cells. MicroRNAs inhibit the transcription or translation of mRNA with which they interact (Paul et al., 2021). Many studies have reported regulatory roles of miRNA in multiple biological processes, including cell proliferation, cell death, cell differentiation, and tumorigenesis (Osaki et al., 2015; Wang et al., 2017; Terlecki-Zaniewicz et al., 2018). Let-7, miR-200, the miR-30 family, and the miR-17-92 gene cluster are reported to be involved in pregnancy-related functions (Liu F. et al., 2016). Kang et al. (2015) found that miR-145 could prevent the attachment of embryos to the endometrium by reducing the expression of insulin-like growth factor 1 receptor (IGF1R) in the endometrium of mice. Although it has been shown that hormones, miRNAs, and transcription factors participate in RM by regulating proliferation and apoptosis (Meng et al., 2021), the mechanisms underlying RM have yet to be identified clearly. Furthermore, the upstream molecular mechanism of miRNA in RM is still unclear.

Circular RNAs (CircRNAs) are circular RNA molecules with closed loops formed by reverse splicing of pre-messenger RNA (pre-mRNA) (Hsiao et al., 2017). They are highly conserved in evolution and abundantly expressed and exhibit tissue, developmental stage, and disease-specific expression patterns (Jin et al., 2016). CircRNAs often act as competitive endogenous RNAs (ceRNAs), upregulating the expression of target miRNA by a sponge-like mechanism (Zheng et al., 2016). According to the study by Zheng et al. (2016), circ-calm4 acts as a miR-337-3p sponge to regulate the level of Myosin 10 (Myo10), promoting the proliferation of smooth muscle cells in the pulmonary artery (Zhang et al., 2020). Many studies have shown that circRNAs act upstream of miRNAs and play important roles in the pathogenesis of many diseases, including esophageal squamous cell carcinoma (Li et al., 2015), colorectal cancer (Zheng et al., 2016; Xu et al., 2017), malignant glioma (Zheng et al., 2017), and head and neck squamous cell carcinoma (Verduci et al., 2017). Recent studies have associated circRNAs with gametogenesis, early embryonic development, embryo implantation, preeclampsia, habitual abortion, and endometriosis (Ge et al., 2019; Zhang et al., 2019; Li Z. et al., 2020). For example, circ-8073 has been shown to regulate goat embryo implantation by targeting miR181a-neurotensis and inhibiting epithelial cell apoptosis (Zhang et al., 2019). Circ-3175-miRr182-testin participates in the development of goat endometrial receptivity (Zhang et al., 2018). Li Z. et al. (2020) found that circ-ZUFSP regulates trophoblast migration and invasion by sponging miR-203 to regulate STOX1 expression. In addition, Zhu et al. reported the regulatory effect of circular RNA PUM1 (CircPUM1) on trophoblast cell dysfunction and inflammation in recurrent spontaneous abortion (Zhu et al., 2021). However, the roles of circRNAs in the decidua of patients with early RM are largely unknown.

In a previous study, microarrays were used to identify differentially expressed circRNAs and mRNAs in the decidua of patients with early RM and an unintended pregnancy control group. We identified 78 upregulated and 45 downregulated circRNAs, as well as 109 upregulated and 97 downregulated mRNAs (fold change ≥ 1.5 and *P*-value < 0.05). Expression of circ-CYP24A1 was significantly higher in the decidua of patients with early RM. Considering the endogenous competitive effect of circRNA, a circRNA-miRNA-mRNA interaction network was constructed using bioinformatics analyses of the microarray data. The miR-224-prolactin receptor (PRLR) axis was investigated to predict the downstream molecular mechanisms. PRLR plays an important role during pregnancy as the receptor of PRL (Kelly et al., 2001, 2002). Besides, Zhang et al. (2021) reported that downregulation of circ-CYP24A1 inhibited cell proliferation, migration, and invasion significantly, while cell apoptosis was enhanced. The timely proliferation and apoptosis of endometrium are critical issues in the establishment of pregnancy. Therefore, the functions of circ-CYP24A1 and its potential role in RM were investigated further in this study.

## Materials and Methods

### Subjects and Decidua Collection

We enrolled 10 healthy pregnant women with unintended pregnancy as the control group and 12 early RM women as the experimental group, with early RM defined as at least two consecutive unexplained miscarriages with regular menstrual cycles. Both groups were enrolled at the Department of Family Planning at the Chongqing Health Centre for Women and Children (Chongqing, China) from December 2016 to September 2017. The mean ages of the control group and the experimental group were 30.5 ± 3.1 and 30.8 ± 3.1 years, respectively, with mean gestational ages of 7.50 ± 0.58 and 7.75 ± 0.50 months, respectively. Patients with infections, endocrine/metabolic disorders, anatomical abnormalities, autoimmune diseases, or parental chromosomal abnormalities were excluded. All participants provided informed consent to have their clinical samples used in this study. All patients included in this study provided written consent before surgery, and the Institutional Review Board for Ethics of Chongqing Medical University and Chongqing Health Centre for Women and Children approved the study protocol. Decidua tissues were separated from the products of conception immediately, thoroughly washed with sterile normal saline, and stored in liquid nitrogen for future use.

### Cell Culture and Transfection

Ishikawa cells were obtained from the American Type Culture Collection (ATCC) and cultured in RPMI-1640 with 5% fetal bovine serum (FBS) and 1% penicillin/streptomycin (Beyotime, Shanghai, China). The medium was replenished every 48 h. The human endometrial stromal cell lines (CRL-4003) were cultured in Dulbecco’s modified Eagle’s medium (DMEM)/Ham’s F-12 medium (Sigma-Aldrich, Saint Louis, MO, United States) supplemented with 10% charcoal-stripped FBS (Biological Industries, Israel) and 1% penicillin/streptomycin (Beyotime, Shanghai, China). All cells were cultured in a 5% CO_2_ incubator at 37°C. The circ-CYP24A1 siRNA, miR-224 mimic, miR-224 inhibitor, and *PRLR* siRNA were transfected using Lipofectamine 2000 (Lip 2000; Invitrogen, Carlsbad, CA, United States). The cells were cultured for 2 h in a penicillin/streptomycin-free medium before transfection. SiRNA and Lip 2000 were diluted separately in 50 μl RPMI-1640 or DMEM/Ham’s F-12 medium and mixed for 15 min at 25°C away from light. Equal amounts of components were then added to orifice plates, and the penicillin/streptomycin medium was added to make the total volume of 500 μl. The circ-CYP24A1, *PRLR*, and negative control (NC) siRNAs were purchased from Sangon Biotech (Shanghai, China). The miR-224 mimic, miR-224 inhibitor, and NC were purchased from RuiBo Biotechnology (Guangzhou, China).

### *In vitro* Decidualization

*In vitro* artificially induced decidualization of human endometrial stromal cell lines was achieved as described earlier (Gellersen and Brosens, 2014; Salsano et al., 2017). Cells were cultured in 2.5% complete medium containing medroxyprogesterone 17-acetate (MPA; 1 mM) and the stable cyclic adenosine 3′:5′ monophosphate (cAMP) analog 8-bromo-cAMP (0.5 mM). The induction for *in vitro* decidualization took 7 days, and the culture medium was changed every 2 days. The circ-CYP24A1 siRNA and miR-224 inhibitor were transfected after artificially induced decidualization *in vitro*.

### Cell Counting Kit-8 Assay

Cell counting kit-8 cell (CCK-8) counting kit (Beyotime Biotechnology, Nanjing, China) was used to detect cell proliferation rates. The Ishikawa cells were plated in 96-well plates at a density of 1 × 10^4^ per well and cultured for 24 h. As mentioned earlier, siRNA and inhibitor experiments were conducted. The effect of SiRNA and inhibitor on the proliferation of Ishikawa cells was assessed every 24 h for 72 h after transfection. A 10 μl CCK-8 solution was added to each well at the specified time points, following incubation for 1 h at 37°C. The absorbance at 450 nm was calculated using a microplate reader (Multiskan FC, Thermo, Waltham, MA, United States). All experiments were performed in triplicate.

### Dual-Luciferase Assay

According to the bioinformatics software (circinteractome, miRanda, and TargetScan), we predicted the binding sties between circ-CYP24A1 and miR-224, as well as the potential target genes of miR-224. Predicted binding sites between circ-CYP24A1 and miR-224, miR-224 and *PRLR*, and a mutant sequence that lack the binding site were cloned into GV272, which was purchased from JiKai Biotechnology (Shanghai, China). These constructs and the miR-224 mimic were co-transfected into HEK293T cells cultured in a 96-well plate. After 48 h of incubation, Firefly and Renilla luciferase activities were measured using the Promega Dual-Luciferase system (Shanghai Genechem Co., Ltd., Shanghai, China), according to the manufacturer’s instructions. To lyse cells, 100 μl of Passive Lysis Buffer was added to 96-well plate, and the Lysis Solution was collected. Then, Firefly luciferase was calculated by adding 100 μl Luciferase Assay Reagent II and 20 μl collected cell Lysis Solution into a 96-well plate. The Renilla fluorescence was calculated by adding 100 μl Luciferase Assay Reagent to the 96-well plate. Relative luciferase activity was calculated as the ratio of Firefly fluorescence to Renilla fluorescence. According to the bioinformatics software (CircInteractome, miRanda, and TargetScan), we predicted the binding sties between circ-CYP24A1 and miR-224, as well as the potential target genes of miR-224. Predicted binding sites between circ-CYP24A1 and miR-224, miR-224 and PRLR, and a mutant sequence lacking the binding site were cloned into GV272, which was purchased from JiKai Biotechnology (Shanghai, China). These constructs and the miR-224 mimic were co-transfected into HEK293T cells cultured in a 96-well plate. After 48 h of incubation, Firefly and Renilla luciferase activities were measured using the Promega Dual-Luciferase system (Shanghai Genechem Co., Ltd., Shanghai, China), according to the manufacturer’s instructions. HEK293T cells were lysed with Passive Lysis Buffer, and then Luciferase Assay Reagent II was added to the cell Lysis Solution to obtain and record the Firefly luciferase. Then Stop & Glo^®^ Reagent was added to the plate to get the Renilla fluorescence. Relative luciferase activity was calculated as the ratio of Firefly fluorescence to Renilla fluorescence.

### Real-Time PCR

As per the manufacturer’s protocol, total RNA was extracted using the RNAiso Plus reagent (TaKaRa, Kyoto, Japan) and reverse transcribed using the PrimeScript™ RT kit (TaKaRa, Kyoto, Japan). The RNA expression was calculated on the Bio-Rad CFX Manager Detection system (United States) using the SYBR Premix Ex Taq™ kit (TaKaRa, Kyoto, Japan). The RNA expression was calculated using the 2^–ΔΔ CT^ method. *GAPDH* was used as an internal control for mRNA and circRNA. U6 was used as an internal control for miRNA. The primers for circ-CYP24A1 were designed by Guangzhou Jisai Co. Ltd. (Guangzhou, China) and synthesized by Sangon Biotech (Shanghai, China). The primer for miR-224 was designed and synthesized by Sangon Biotech (Shanghai, China). PCR primers (Sangon Biotech, Shanghai, China) are shown in Table 1.

**TABLE 1:** Primer sequences.

Genes	Sequence
β-actin (Human)	F: 5′-GTGGCCGAGGACTTTGATTG-3′ R: 5′-CCTGTAACAACGCATCTCATATT-3′
circ-CYP24A1	F: 5′-TGAGCCTGTTGAGATGCTACAC-3′ R: 5′-TGGTACTCCACCTGAGGCG-3′
IGFBP1	F: 5′-ATCAGCCCATCCTGTGGAAC-3′
	F: 5′-TGCAGCTAATCTCTCTAGCACTT-3′
miR-224	F:5′-TCAAGTCACTAGTGGTTCCGTTTAG-3′

### *In situ* Hybridization

Digoxygenin-labeled probes specific for circ-CYP24A1 were designed and synthesized by Guangzhou Jisai Co. Ltd. (Guangzhou, China) As described in the previous study of our team (Liu S. et al., Reprod Sci., 2014, 21:686–695), ISH was performed as follows. Frozen tissue sections (10 μm) were prepared and fixed on glass slides pre-treated with poly-lysine. After being fixed in 4% paraformaldehyde and treated with 1% Triton X-100, sections were incubated with the digoxin-labeled probes at 42°C for 16 h for hybridization, using an anti-digoxygenin antibody conjugated to alkaline phosphatase (1:5,000, Roche, Basel, Swiss Confederation). The NC was incubated with hybridization fluid instead of probes. Nitro-blue-tetrazolium and 5-bromo-4-chloro-3-indolyl-phosphate (Beyotime Biotechnology, Nanjing, China) were then used for staining. The nuclei were counterstained with 1% methyl green. Finally, sections were photographed using a photomicroscope (Olympus, Tokyo, Japan).

### Immunohistochemistry

As described in a previous study (Su et al., 2020), IHC was performed as follows. Uterine tissue samples were fixed in a 4% paraformaldehyde solution for 4–6 h, dehydrated with increasing concentrations of ethanol (75, 85, 95, and 100%), and embedded in paraffin. Sections of 5 μm were cut for further detection. Antigen retrieval was carried out in pH 8.0 EDTA buffer (Zhongshan Biosciences, Beijing, China) for 15 min at 95°C, followed by cooling naturally to room temperature. H_2_O_2_ (3%) was used to eliminate the endogenous peroxidase interference. To block non-specific binding, sections were incubated with 10% goat serum and then with the mouse monoclonal anti-PRLR (1:500; Abcam, Cambridge, United Kingdom) overnight at 4°C. The NC was incubated with isotype IgG (1:500; Bioss, Beijing, China) instead of primary antibody. Isotype IgG is used at the same concentration as the primary antibody. The sections were then incubated with secondary antibodies at 37°C for 1 h, followed by an avidin-biotinylated peroxidase complex system (Zhongshan Biosciences, Beijing, China) for 30 min. The chromogenic reaction was initiated by incubating with diaminobenzidine (Zhongshan Biosciences, Beijing, China) for 3–5 min and terminated with water. Nuclei were counterstained with hematoxylin. Finally, sections were photographed using a photomicroscope (Olympus, Tokyo, Japan).

### Immunofluorescence

The Ishikawa cells were cultured in a 48-well plate with coverslips. When the density of adherent cells reached about 80%, the cell slides were fixed with ice methanol for 15 min, and PBS was washed three times for 3 min each time. The cells were perforated by incubating in a 0.5% Triton X-100 solution for 20 min at room temperature. After washing for three times, the cell slides were incubated with the mouse monoclonal anti-Ki67 (1:500; 9449T; Cell Signaling Technology, Boston, MA, United States) overnight at 4°C. The slides were washed and incubated with a CY3-labeled fluorescent mouse antibody for 1 h at 37°C. The plate was incubated with DAPI for 10 min and sealed with anti-fluorescence quenching tablets. Finally, the image was captured using a microscope and quantified using ImageJ software.

### Terminal Deoxynucleotidyl Transferase-Mediated dUTP-Biotin Nick End Labeling Assay

A one-step terminal deoxynucleotidyl transferase-mediated dUTP-biotin nick end labeling (TUNEL) assay kit (Beyotime Biotechnology, Nanjing, China) was used to detect apoptosis in Ishikawa cells. The Ishikawa cells were plated in 48-well plates at a density of 1 × 10^4^ per well and cultured for 24 h. siRNA and miR-224 inhibitor experiments were conducted as mentioned earlier. The cells were fixed with 4% paraformaldehyde and permeabilized with 0.3% Triton X-100. The cells were then incubated at 37°C for 60 min in the dark with TUNEL reaction buffer according to the manufacturer’s protocol. Subsequently, fluorescence microscopy was used to observe the apoptotic cells.

### Western Blotting

Total proteins were extracted using a protein extraction kit purchased from Beyotime (Shanghai, China). The extracts were fractionated on 10% sodium dodecyl sulfate-polyacrylamide gels and then transferred to polyvinylidene difluoride membranes (Millipore, Billerica, MA, United States). To block non-specific binding, membranes were incubated in 5% BSA and then incubated overnight with primary antibodies. The primary antibodies used were as follows: mouse monoclonal anti-PRLR (1:500; ab2773, Abcam, Cambridge, United Kingdom), mouse monoclonal anti-cyclinD3 (1:200; 2936 s, Cell Signaling Technology, Boston, MA, United States), mouse monoclonal anti-PCNA (1:500; 2586 s; Cell Signaling Technology, Boston, MA, United States), rabbit polyclonal anti-Bax (1:300; 2772 s; Cell Signaling Technology, Boston, MA, United States), and mouse monoclonal anti-Bcl-2 (1:300; 3498 s; Cell Signaling Technology, Boston, MA, United States). After incubating with secondary antibodies, antibody binding was quantified using an ECL reagent (Millipore, Darmstadt, Germany), and bands were visualized using a ChemiDoc™ XRS + (Bio-Rad).

### Statistical Analysis

All data are shown as means ± SEM. All experiments were repeated at least three times. Data were analyzed using the SPSS 22.0 package (SPSS China, Shanghai, China). Comparisons between two groups were calculated using *t*-tests. Comparisons among three or more groups were calculated using one-way ANOVA. The *p*-value < 0.05 was considered statistically significant.

## Results

### Circ-CYP24A1 Acts as a Sponge for miR-224

**FIGURE 1:**
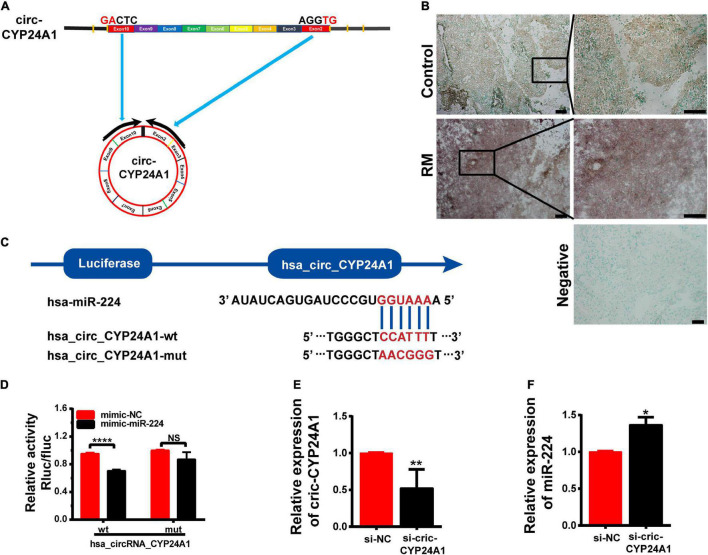
Circ-CYP24A1 act as a sponge of miR-224. **(A)** Circ-CYP24A1 was derived from Exon 3 to Exon 10 of the human CYP24A1 gene. **(B)** *In situ* hybridization staining of circ-CYP24A1 in the decidua of patients with early RM and unintended pregnant women. Scale bar: 100 μm. **(C)** Bioinformatics prediction of the binding site of miR-224 to circ-CYP24A1 and the wild and mutant sequences of circ-CYP24A1 luciferase plasmid. **(D)** Expression of luciferase activity. **(E,F)** Circ-CYP24A1 and miR-224 levels were detected using RT-PCR. RM, early recurrent miscarriage; control, unintended pregnant women; si-NC, negative control to siRNA (*p < 0.05, **p < 0.01, ****p < 0.001).

Circular CYP24A1 was derived from the segment spanning exons 3–10 of the *CYP24A1* gene of human chromosome 20. Circ-CYP24A1 is located at chr20:52773707-52789638 with a length of 1297 nt (Figure 1A). Salzman et al. (2013) had reported the relative expression of Circ-CYP24A1 in A549, AG04450, and HeLa cells. However, the function of Circ-CYP24A1 is still not clear. CircRNAs often function as ceRNAs to adsorbed miRNAs, thus regulating mRNA and protein synthesis. In our previous study, using bioinformatics predictions from miRanda and the TargetScan database, we found that circ-CYP24A1 may act as a sponge for miR-224 and regulate the expression of the *PRLR* gene. We had demonstrated high expression of circ-CYP24A1 and *PRLR* and decreased expression of miR-224 in the decidua of patients with early RM (Li C. et al., 2020). In this study, we used ISH to verify the expression of circ-CYP24A1 in the decidua of patients with early RM and unintended pregnant women. As shown in Figure 1B, the high expression of circ-CYP24A1 in the decidua of patients with an early RM was observed, which is consistent with our previous data. To analyze the binding of miR-224 and circ-CYP24A1, a luciferase reporter assay was conducted. As shown in Figure 1C, a fragment of circ-CYP24A1, including the predicted target site or a mutated target site, was inserted into the downstream part of the Firefly luciferase gene (pmirGLO-circ-CYP24A1-wt and pmirGLO-circ-CYP24A1-mut). The plasmids were co-transfected with miR-224 mimic or mimic NC into HEK293T cells. Overexpression of miR-224 significantly reduced luciferase activity in cells transfected with the vector containing the complete circ-CYP24A1 sequence but did not change the luciferase activity with the vector containing the mutated miR-224 binding site (Figure 1D). The result suggests that circ-CYP24A1 could act as a molecular sponge for miR-224. To verify the competing endogenous function of circ-CYP24A1, the expression of miR-224 was examined after circ-CYP24A1 silencing. The transfection efficiency was verified using Real-time PCR (RT-PCR) (Figure 1E). As shown in Figure 1F, relative to the NC, the level of miR-224 increased significantly in Ishikawa cells transfected with circ-CYP24A1 siRNA.

### PRLR Is a Downstream Target of the Circ-CYP24A1-miR-224 Axis

**FIGURE 2:**
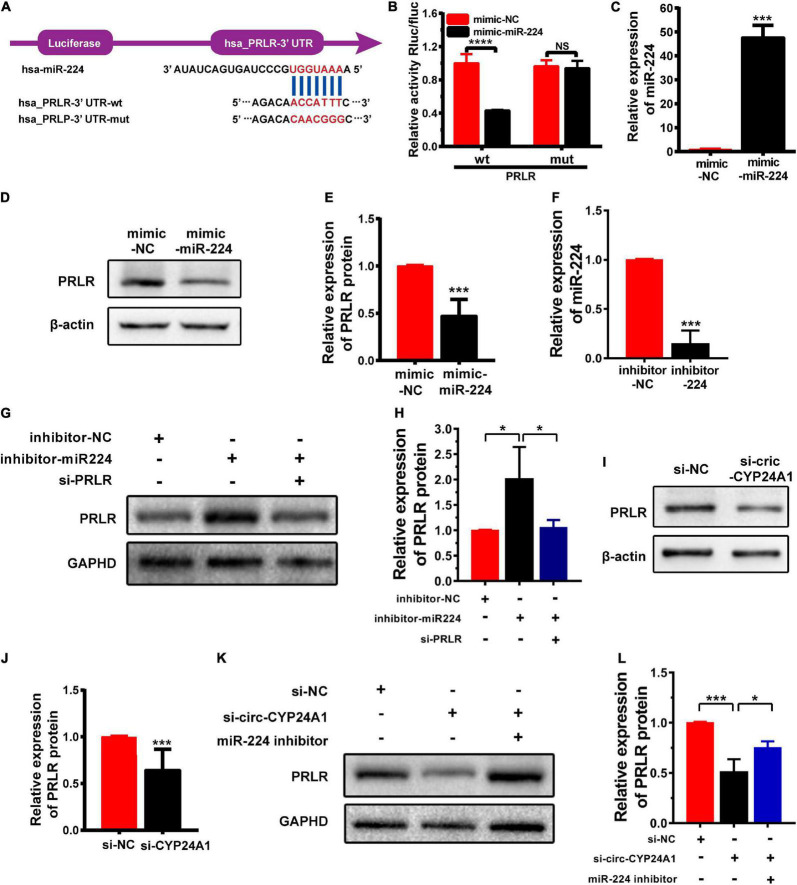
PRLR is a downstream target of the circ-CYP24A1-miR-224 axis. **(A)** Bioinformatics prediction of the binding site of miR-224 to PRLR mRNA 3′ UTR and the wild and mutant sequences of PRLR mRNA 3′ UTR luciferase plasmid. **(B)** Expression of luciferase activity. **(C)** miR-224 levels were detected using RT-PCR. **(D)** The expression of PRLR was detected in Ishikawa cells transfected with miR-224 mimic. **(E)** Quantitative analysis of D. **(F)** miR-224 levels were detected using RT-PCR. **(G)** The expression of PRLR was detected in Ishikawa cells either transfected with miR-224 inhibitor or co-transfected with miR-224 inhibitor and PRLR siRNA. **(H)** Quantitative analysis of G. **(I)** The expression of PRLR was detected in Ishikawa cells transfected with circ-CYP24A1 siRNA. **(J)** Quantitative analysis of I. **(K,L)** The expression of PRLR was detected in Ishikawa cells transfected with circ-CYP24A1 siRNA or co-transfected with circ-CYP24A1 siRNA and miR-224 inhibitor. si-NC, negative control to siRNA; mimic-NC, negative control to biological mimic; inhibitor-NC, negative control to miR-224 inhibitor (*p < 0.05, ***p < 0.005, ****p < 0.001).

Circular RNAs act as miRNA sponges to regulate their target genes (Hsiao et al., 2017). In this study, we verified whether *PRLR* was the downstream target of the circ-CYP24A1-miR-224 axis. The 3′ UTR fragments of PRLR mRNA containing miR-224 binding sites and the same fragments with mutated binding sites were cloned into the pGL3-basic luciferase reporter vectors (Figure 2A). As shown in Figure 2B, the co-transfection of pmirGLO-PRLR-wt and miR-224 mimic into HEK293T cells reduced luciferase activity compared with the co-transfection of pmirGLO-PRLR-mt and mimic NC. While no significant difference was observed in group co-transfection of pmirGLO-PRLR-mut and miR-224 mimic and group co-transfection of pmirGLO-PRLR-mut and mimic NC, this assay confirmed the direct binding of miR-224 and *PRLR* mRNA 3′ UTR. The regulatory effect of miR-224 on PRLR was further confirmed using the miR-224 mimic to simulate the high expression of miR-224 in Ishikawa cells. The stimulatory effect of miR-224 mimic was confirmed using RT-PCR (Figure 2C). The expression of PRLR was significantly decreased after miR-224 mimic treatment (Figures 2D,E). Besides, a rescue experiment was further conducted in Ishikawa cells. Ishikawa cells were transfected with miR-224 inhibitor or co-transfected with miR-224 inhibitor and PRLR siRNA. The transfection efficiency was verified using RT-PCR (Figure 2F). The expression of PRLR was significantly increased after miR-224 inhibitor treatment. While the activation of miR-224 on PRLR expression was significantly inhibited by PRLR siRNA (Figures 2G,H), considering that decidual cells are more representative cells model of decidua tissue, we conducted the rescue experiment in decidual cells. First, the human endometrial stromal cell line was induced to transform into the decidual cell using medroxyprogesterone 17-acetate (MPA; 1 mM) and stable cyclic adenosine 3′:5′ monophosphate (cAMP) analog 8-bromo-cAMP (0.5 mM) for 7 days. High expression of IGFBP1 mRNA indicated the decidualization of the stromal cell (Supplementary Figure 1B). The decidual cells were then transfected with miR-224 inhibitor or co-transfected with miR-224 inhibitor and PRLR siRNA. The results were similar to that in Ishikawa cells (Supplementary Figures 1C,D). Next, the expression of PRLR protein after circ-CYP24A1 silencing was examined using Western blotting. As shown in Figures 2I,J, compared with the NC (si-NC), PRLR expression was significantly reduced in the Ishikawa cell treated with circ-CYP24A siRNA. To confirm that circ-CYP24A regulates PRLR expression through sponging miR-224, a rescue experiment was conducted in both Ishikawa cells and decidual cells. As shown in Figures 2K,L, and Supplementary Figures 1E,F, the expression of PRLR reduced significantly after circ-CYP24A1 silencing, and the miR-224 inhibitor reversed the suppression of circ-CYP24A1 siRNA on PRLR expression. Overall, these results indicated that circ-CYP24A1 elevates PRLR expression by acting as a sponge for miR-224.

### Circ-CYP24A1 Regulates Cell Proliferation and Apoptosis *via* the miR-224_PRLR Axis

**FIGURE 3:**
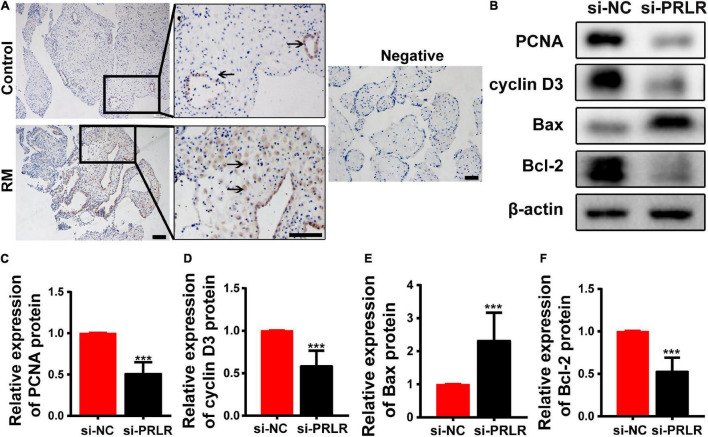
Circ-CYP24A1 regulated cell proliferation and apoptosis *via* the miR-224_PRLR axis. **(A)** Expression of PRLR was detected using immunohistochemical staining in the decidua of patients with early RM and unintended pregnant women. **(B)** Expression of proliferation-related markers was detected using Western blotting in Ishikawa cells transfected with PRLR siRNA. **(C–F)** Gray analysis of B. RM, early recurrent miscarriage; control, unintended pregnant women; si-NC, negative control to siRNA (***p < 0.005). Scale bar: 100 μm.

Circular RNAs often act as miRNA sponges, regulating mRNAs that function in the cell cycle (Du et al., 2016), apoptosis (Du et al., 2017a), and senescence (Du et al., 2017b). Defects in the proliferation and apoptosis of the decidual cells are known as two important issues associated with RM. IHC was performed to verify the levels and locations of PRLR protein expression. As shown in Figure 3A, high expression was observed in the decidua of patients with early RM. PRLR has been reported to promote cell proliferation and inhibit apoptosis in breast cancer (Leehy et al., 2018). In this study, the proliferation and apoptosis of Ishikawa cells were detected, even after PRLR silencing. As shown in Figures 3B–F, knocking out PRLR significantly decreased the expression of the proliferation-related proteins Cyclin D3 and PCNA. Simultaneously, enhanced expression of Bcl-2 and reduced expression of Bax were detected after *PRLR* silencing, reflecting the activation of apoptosis. The above results demonstrated the role of PRLR in promoting proliferation and inhibiting apoptosis.

**FIGURE 4:**
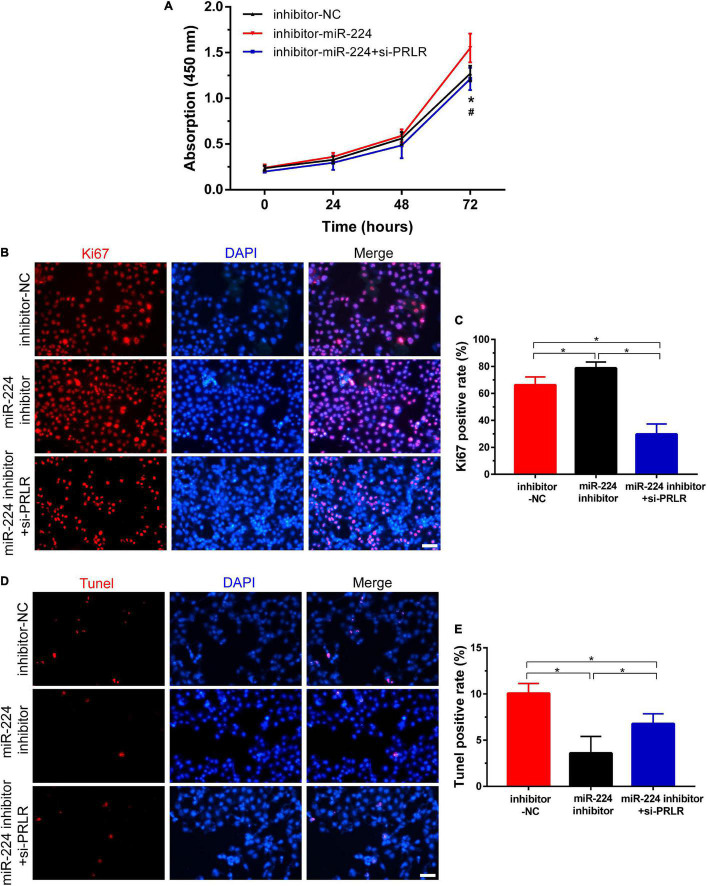
Circ-CYP24A1 regulated cell proliferation and apoptosis *via* the miR-224-PRLR axis. **(A)** Cell viability was detected in Ishikawa cells transfected with miR-224 inhibitor or co-transfected with miR-224 inhibitor and PRLR siRNA using CCK8 assay. **(B)** Immunofluorescence staining for Ki67 in Ishikawa cells. **(C)** Quantitative group data for Ki67 positive cells. **(D)** Apoptosis of Ishikawa cells was tested using the TUNEL assay. **(E)** Quantitative group data for TUNEL positive cells. inhibitor-NC, negative control to miR-224 inhibitor; #, comparison between miR-224 inhibitor and miR-224 inhibitor + si-PRLR (*p < 0.05, #*p* < 0.05). Scale bar: 100 μm.

As reported earlier, miR-224 plays an important role in regulating the proliferation and apoptosis of many diseases (Liu W. et al., 2016; Liu et al., 2020; Xie et al., 2020). In this study, CCK-8 assay and IF staining were used to verify the role of miR-224 in regulating proliferation in Ishikawa cells. Ishikawa cells were treated with miR-224 inhibitor or co-transfected with miR-224 inhibitor and PRLR siRNA. Cell viability was then detected at different time points. As shown in Figure 4A, miR-224 inhibitor showed a significant increase in cell viability compared with control (inhibitor-NC). The increase in cell viability was inhibited by PRLR siRNA. The IF staining of Ki67 yielded similar results, the miR-224 inhibitor significantly increased the expression of Ki67 in Ishikawa cells, and PRLR siRNA suppressed the activation of Ki67 (Figures 4B,C). Then TUNEL assay was used to verify the regulating function of miR-224 in apoptosis in Ishikawa cells. TUNEL-positive cells were observed in the inhibitor-NC, miR-224 inhibitor, and miR-224 inhibitor + si-PRLR groups (Figures 4D,E). Apoptosis of Ishikawa cells was decreased in the miR-224 inhibitor group compared with the control group. However, cell apoptosis in the miR-224 inhibitor + si-PRLR group was increased compared with the miR-224 inhibitor group (Figures 4D,E). These results demonstrated that miR-224 promotes the proliferation and inhibited apoptosis of Ishikawa cells by downregulating PRLR.

**FIGURE 5:**
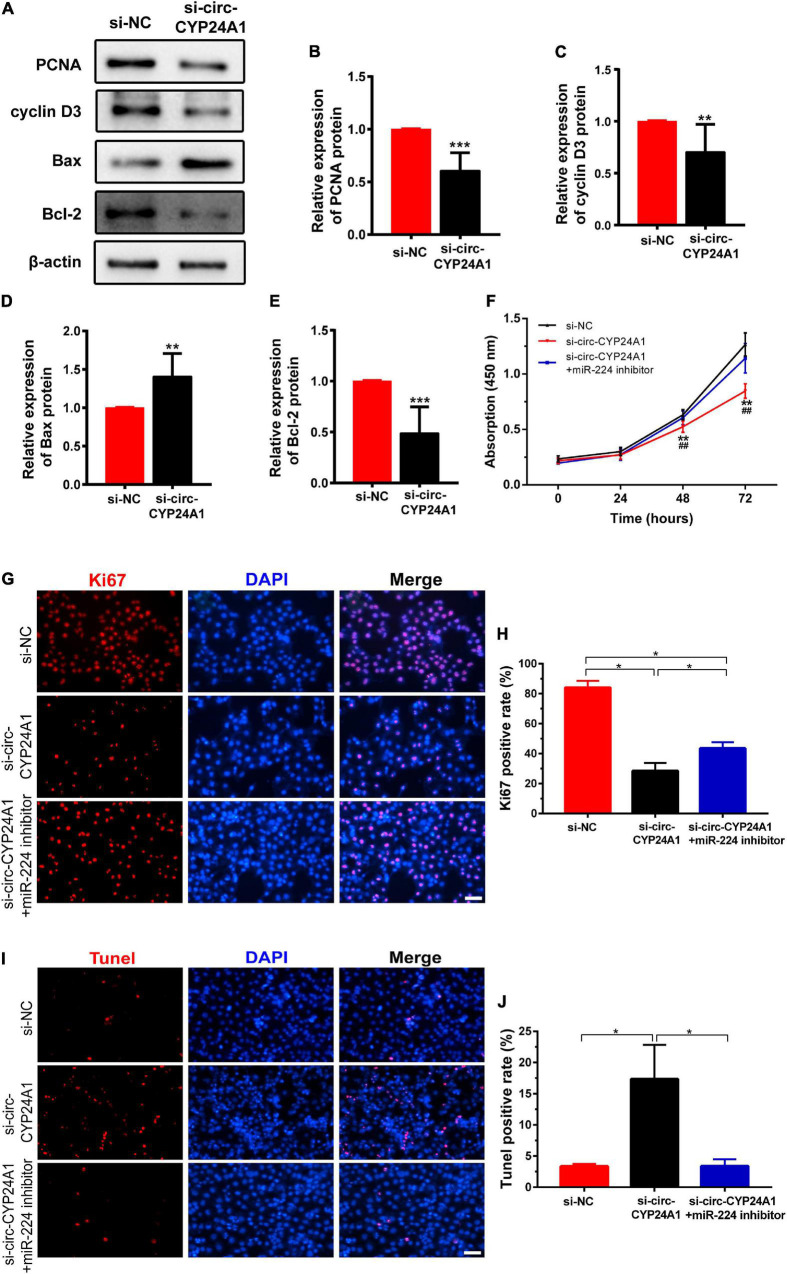
Circ-CYP24A1 regulated cell proliferation and apoptosis *via* the miR-224_PRLR axis. **(A)** Expression of proliferation-related markers was detected using Western blotting in Ishikawa cells transfected with circ-CYP24A1 siRNA. **(B–E)** Gray analysis of A. **(F)** Cell viability was detected in Ishikawa cells transfected with circ-CYP24A1 siRNA or co-transfected with circ-CYP24A1 siRNA and miR-224 inhibitor using the CCK8 assay. **(G)** Immunofluorescence staining for Ki67 in Ishikawa cells. **(H)** Quantitative group data for Ki67 positive cells. **(I)** Apoptosis in Ishikawa cells was tested using the TUNEL assay. **(J)** Quantitative group data for TUNEL positive cells. RM, early recurrent miscarriage; control, unintended pregnant women; si-NC, negative control to siRNA; #, comparison between si-circ-CYP24A1 and si-circ-CYP24A1 + miR-224 inhibitor (**p < 0.01, ***p < 0.005, ##*p* < 0.01). Scale bar: 100 μm.

Next, we investigated the effects of circ-CYP24A1 on proliferation and apoptosis in Ishikawa cells. Western blotting showed that silencing circ-CYP24A1 dramatically reduced the expression of Cyclin D3 and PCNA. Moreover, after circ-CYP24A1 knockout, the expression of Bax was reduced while the expression of Bcl-2 increased significantly (Figures 5A–E). These results indicate that silencing circ-CYP24A1 also increased apoptosis and reduced cell proliferation. Next, we investigated whether circ-CYP24A1 regulates Ishikawa cell proliferation and apoptosis *via* the miR-224-PRLR axis. Ishikawa cells were transfected with circ-CYP24A1 siRNA or co-transfected with circ-CYP24A1 siRNA and miR-224 inhibitor. CCK-8 assay showed that silencing circ-CYP24A1 dramatically reduced cell viability compared with si-NC, while miR-224 inhibitor rescued the reduction of cell viability (Figure 5F). Besides, the expression of Ki67 in Ishikawa cells decreased significantly after silencing circ-CYP24A1, but the reduction was rescued by a miR-224 inhibitor (Figures 5G,H). The level of apoptosis in Ishikawa cells was examined using a TUNEL assay. More TUNEL-positive cells were observed after circ-CYP24A1 knockout. However, compared with the si-circ-CYP24A1 group, the apoptosis was decreased after co-transfection of circ-CYP24A1 siRNA and miR-224 inhibitor (Figures 5I,J). The results above demonstrated that circ-CYP24A1 regulates Ishikawa cell proliferation and apoptosis *via* the miR-224-PRLR axis.

## Discussion

The maintenance of pregnancy is a complex and coordinated process in which non-coding RNAs (ncRNAs) play important roles. The roles of miRNAs during the reproductive process and pregnancy-related diseases have been well established (Liang et al., 2017; Lv et al., 2019). However, few studies have evaluated the roles of circRNAs in patients with early RM. In our previous study, we observed high expression of circ-CYP24A1 and PRLR in the decidua of these patients. Based on our bioinformatics analysis, we hypothesized that circ-CYP24A1 participates in early RM by binding miR-224, thereby regulating the expression of its target gene, *PRLR*. Using luciferase reporter assay, we confirmed the binding of miR-224 with circ-CYP24A12 and miR-224 with PRLR. The expression of miR-224 was significantly reduced in Ishikawa cells treated with circ-CYP24A1 siRNA, which demonstrated that miR-224 was the downstream target of circ-CYP24A1 and could be negatively modulated by circ-CYP24A1. We further found that the expression of PRLR can be activated by miR-224 mimic. The rescue experiment proved that the activation of miR-224 on PRLR can be suppressed by PRLR siRNA. These results demonstrated that PRLR was the downstream target of miR-224 and could be negatively modulated by miR-224. Then, we found that circ-CYP24A1 silencing significantly decreased the expression of PRLR in Ishikawa cells, while miR-224 inhibitor could recover the reduction of PRLR expression. Taken together, these data indicate that circ-CYP24A1 elevates PRLR expression by acting as a sponge for miR-224.

It has been reported that circRNAs are significantly involved in regulating cell proliferation, apoptosis, and differentiation (Jia and Li, 2019; Chen et al., 2021; Gao et al., 2021; Sun et al., 2021). High levels of circRNA_33186 have been reported to promote apoptosis and inhibit proliferation in osteoarthritis (Zhou et al., 2019). CircRNA CBL.11 has been shown to suppress cell proliferation by acting as a sponge to regulate miR-6778-5p function in colorectal cancer (Li et al., 2019). Here, we identified high expression of circ-CYP24A1 in the decidua of patients with early RM. Further analysis indicated that miR-224 and *PRLR* may be the downstream targets of circ-CYP24A1. However, further exploration is required for a more complete understanding of the functional role of circ-CYP24A1 in early RM.

PRLR, as a member of the cytokine receptor superfamily, maintains functional corpus luteum and pregnancy together with PRL. PRLR is expressed in the glandular epithelium and a subset of stromal cells in the decidua (Jones et al., 1998). Herein, high expression of PRLR in the epithelium and stromal cells in decidua was observed using IHC (Figure3A), which was consistent with the previous reports (Kelly et al., 2001, 2002). It has been reported that PRLR-deficient mice exhibited markedly abnormal embryo implantation and decidualization (Kelly et al., 2001, 2002), indicating a functional role for PRLR during pregnancy. Moreover, some researchers reported a positive correlation between PRLR and the proliferation marker Ki67 in the endometrium (Paulson et al., 2020). Multiple studies have also reported that PRLR promotes proliferation (Dandawate et al., 2020).

Considering the abnormal proliferation and apoptosis in RM and the regulatory effect of PRLR on proliferation, functional experiments were further conducted. First, we found that silencing PRLR in Ishikawa cells significantly decreased cell proliferation and enhanced apoptosis. Next, we found that miR-224 showed a significantly downregulating effect on proliferation while upregulating function on apoptosis. In addition, the regulating effect of miR-224 on proliferation and apoptosis could be recovered by PRLR siRNA. These results well demonstrated that miR-224 functioned as upstream of PRLR, regulating proliferation and apoptosis in Ishikawa cells. Moreover, we found that downregulating circ-CYP24A1 in Ishikawa cells also markedly reduced cell proliferation and promoted cell apoptosis. In addition, the regulating effect of circ-CYP24A1 siRNA on proliferation and apoptosis could be recovered by miR-224 inhibitor. These results indicated that circ-CYP24A1 regulated proliferation and apoptosis by binding to and inhibiting miR-224, thereby increasing PRLR expression. During the menstrual cycle, the endometrium undergoes a dynamic process of proliferation, differentiation, apoptosis, and renewal. Estrogen induces endometrial stromal cells proliferation, whereas progesterone blocks proliferation and induces the differentiation of endometrial stromal cells into decidual cells (Kliman and Frankfurter, 2019). The balance and timely transition between proliferation, differentiation, and apoptosis of stromal cells are essential. In this study, our data showed that dysfunction of circ-CYP24A1 impairs this balance between proliferation and apoptosis by regulating miR-224 and PRLR, which may be a new molecular mechanism underlying RM.

In summary, in this study, we demonstrated that circ-CYP24A1 participates in RM. High expression of circ-CYP24A1 may impair the balance of cell proliferation and apoptosis by sponging miR-224, thereby regulating PRLR. This study provided a novel mechanism for RM. However, the specific role of circ-CYP24A1 in proliferation and apoptosis warrants further investigation.

## Data Availability Statement

The original contributions presented in the study are included in the article/Supplementary Material, further inquiries can be directed to the corresponding author/s.

## Ethics Statement

The studies involving human participants were reviewed and approved by Medical Ethics Committee of Chongqing Health Center for Women and Children. The patients/participants provided their written informed consent to participate in this study.

## Author Contributions

YW, RG, and HQ designed the study, interpreted the data, and revised the manuscript. TL and CoL carried out the research. YS analyzed the data and drafted the manuscript. ChL, JX, and XiL helped to collect the tissues and performed the experiments. YD, XC, JH, and XuL assisted in data analysis. All authors contributed to the article and approved the submitted version.

## Conflict of Interest

The authors declare that the research was conducted in the absence of any commercial or financial relationships that could be construed as a potential conflict of interest.

## Publisher’s Note

All claims expressed in this article are solely those of the authors and do not necessarily represent those of their affiliated organizations, or those of the publisher, the editors and the reviewers. Any product that may be evaluated in this article, or claim that may be made by its manufacturer, is not guaranteed or endorsed by the publisher.
