# Supplementary figures and images for: The Circ-CYP24A1-miR-224-PRLR Axis Impairs Cell Proliferation and Apoptosis in Recurrent Miscarriage
Source: Front Physiol. 2022 Mar 3;13:778116. doi: 10.3389/fphys.2022.778116 (PMC8928262; doi:10.3389/fphys.2022.778116)

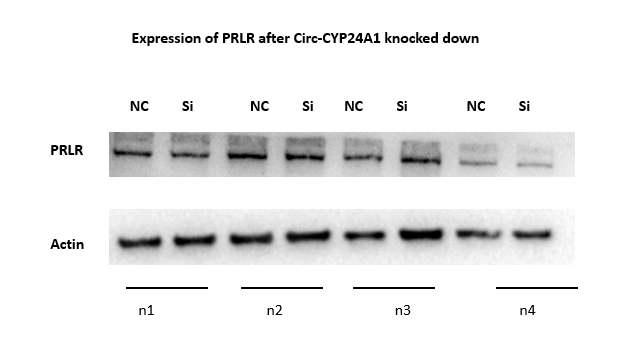


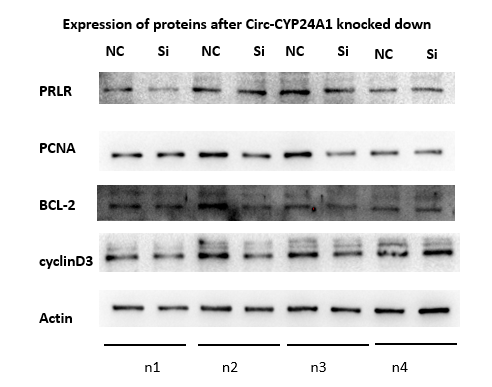


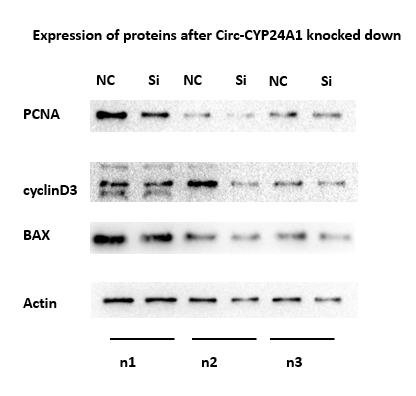


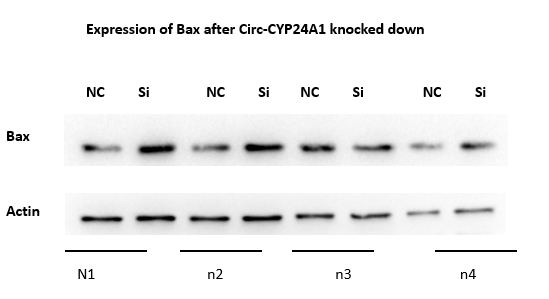


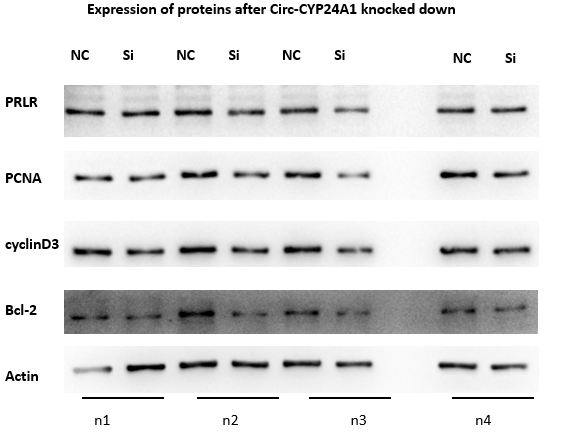


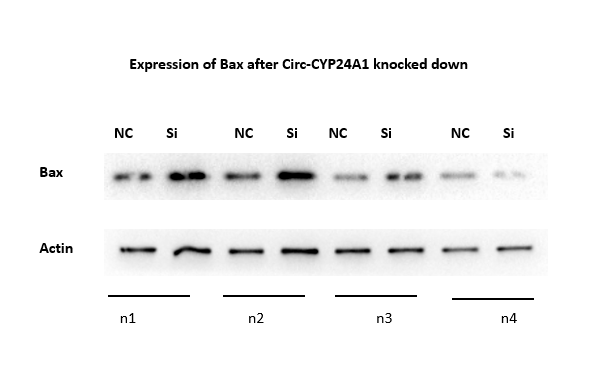


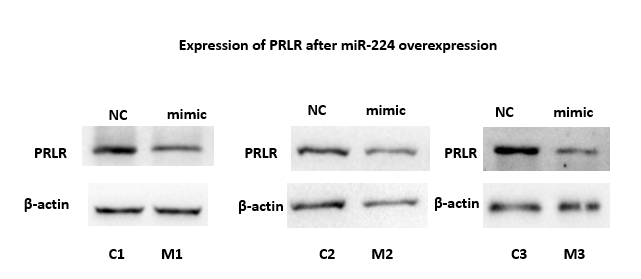


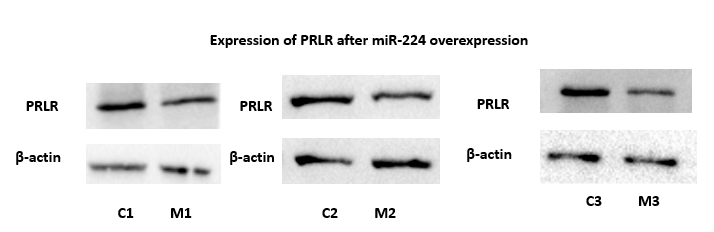


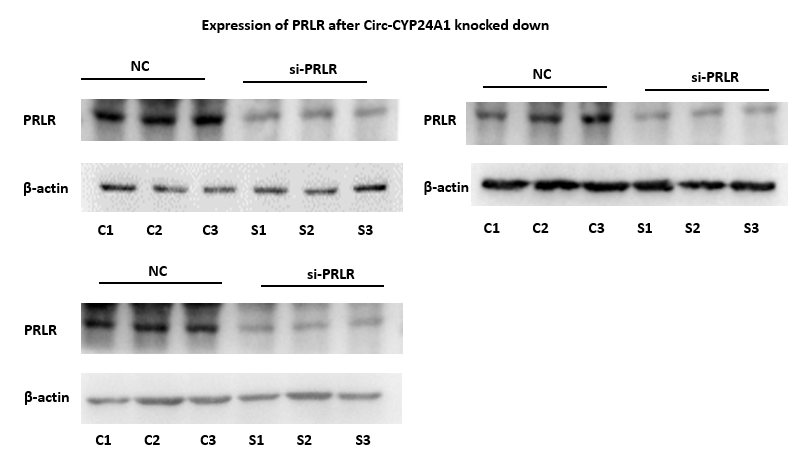


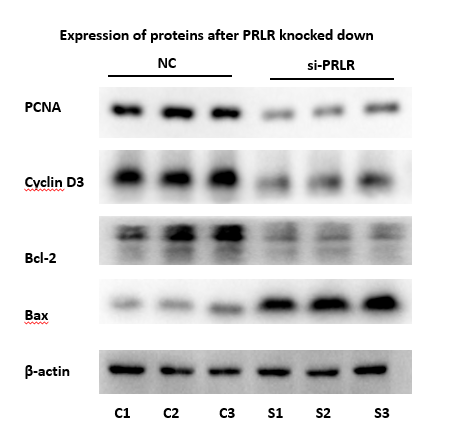


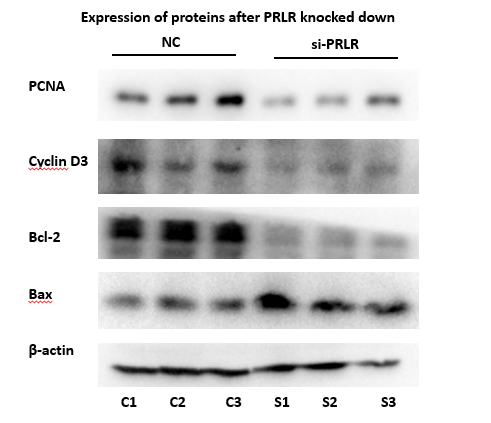


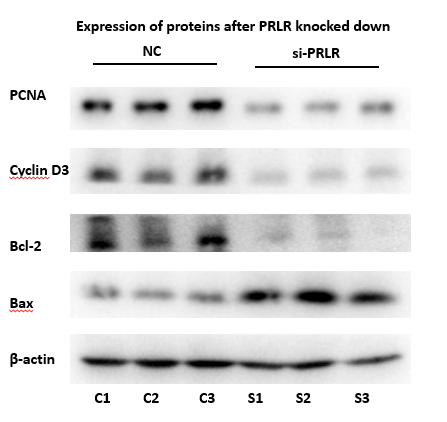

Supplement: Supplementary file 1 [file Data_Sheet_1.DOCX]
