# Supplementary material for: The Circ-CYP24A1-miR-224-PRLR Axis Impairs Cell Proliferation and Apoptosis in Recurrent Miscarriage
Source: Front Physiol. 2022 Mar 3;13:778116. doi: 10.3389/fphys.2022.778116 (PMC8928262; doi:10.3389/fphys.2022.778116)
Supplement: Supplementary file 2 [file Data_Sheet_2.DOC]

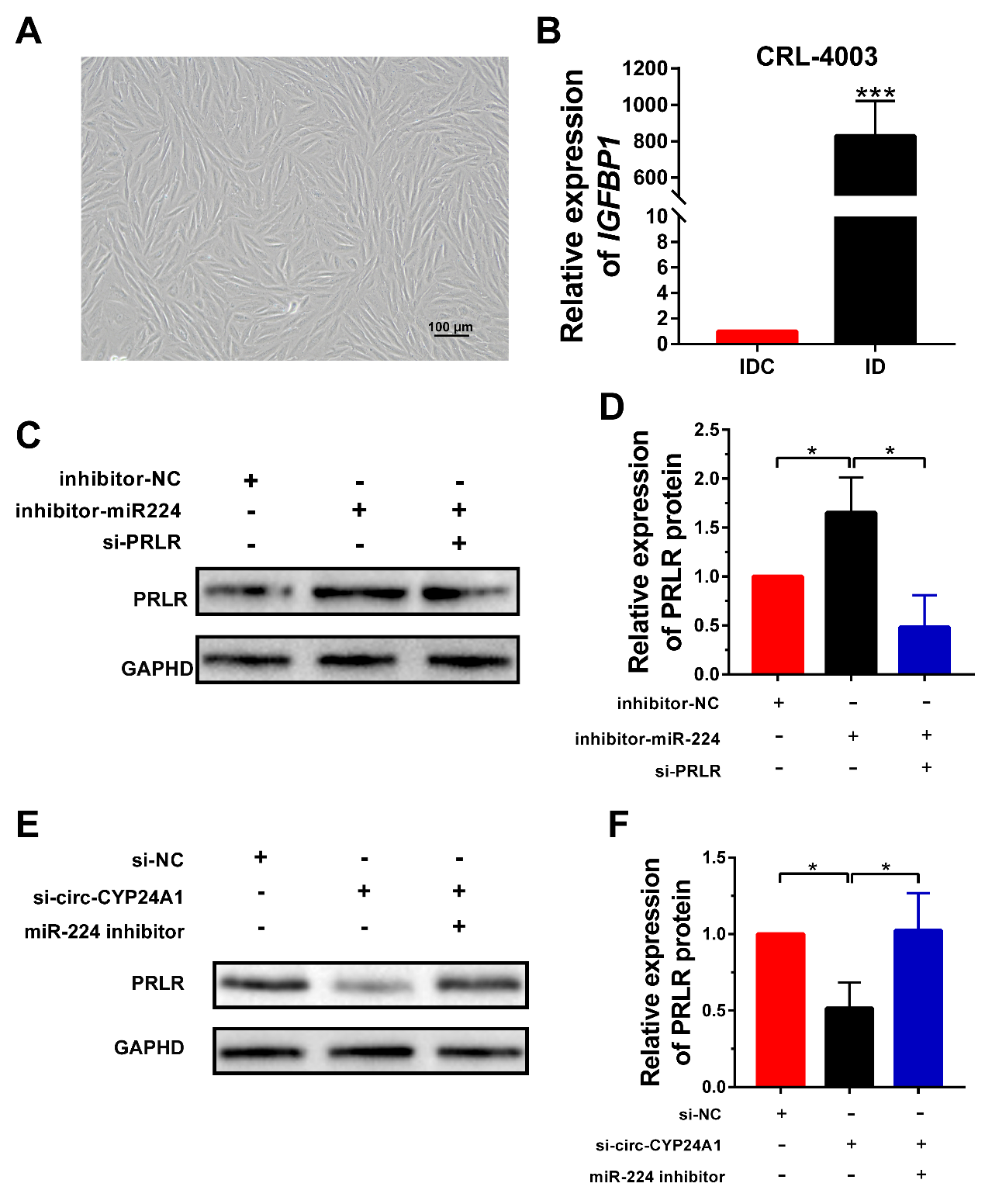


Figure S1 | PRLR is a downstream target of circ-CYP24A1_miR-224 axis in decidual cells. (A) Representative picture of Human endometrial stromal cell line (CRL-4003). (B)The expression of IGFBP1 mRNA was detected by RT-PCR. (C) The expression of PRLR was detected in decidual cells transfected with miR-224 inhibitor or co-transfected with miR-224 inhibitor and PRLR siRNA. (D) Quantitative analysis of C. (E) The expression of PRLR was detected in Ishikawa cells transfected with circ-CYP24A1 siRNA or co-transfected with circ-CYP24A1 siRNA and miR-224 inhibitor.(F) Quantitative analysis of E. si-NC: negative control to siRNA, mimic-NC: negative control to biological mimic, inhibitor-NC: negative control to miR-224 inhibitor, (* p <0.05, *** p <0.005).
